# Liver Cancer: Therapeutic Challenges and the Importance of Experimental Models

**DOI:** 10.1155/2021/8837811

**Published:** 2021-02-28

**Authors:** Marina Galicia-Moreno, Jorge A Silva-Gomez, Silvia Lucano-Landeros, Arturo Santos, Hugo C Monroy-Ramirez, Juan Armendariz-Borunda

**Affiliations:** ^**1**^ University of Guadalajara, Institute of Molecular Biology in Medicine and Gene Therapy, Department of Molecular Biology and Genomics, Health Science University Center (CUCS), Guadalajara 44340, JAL, Mexico; ^**2**^ Tecnologico de Monterrey, Escuela de Medicina y Ciencias de la Salud, Zapopan 45138, Jalisco, Mexico

## Abstract

Liver cancer is one of the main causes of death related to cancer worldwide; its etiology is related with infections by C or B hepatitis virus, alcohol consumption, smoking, obesity, nonalcoholic fatty liver disease, diabetes, and iron overload, among other causes. Several kinds of primary liver cancer occur, but we will focus on hepatocellular carcinoma (HCC). Numerous cellular signaling pathways are implicated in hepatocarcinogenesis, including YAP-HIPPO, Wnt-*β*-catenin, and nuclear factor-*κ*B (NF-*κ*B); these in turn are considered novel therapeutic targets. In this review, the role of lipid metabolism regulated by peroxisome proliferator-activated receptor gamma (PPAR*γ*) in the development of HCC will also be discussed. Moreover, recent evidence has been obtained regarding the participation of epigenetic changes such as acetylation and methylation of histones and DNA methylation in the development of HCC. In this review, we provide detailed and current information about these topics. Experimental models represent useful tools for studying the different stages of liver cancer and help to develop new pharmacologic treatments. Each model *in vivo* and *in vitro* has several characteristics and advantages to offer for the study of this disease. Finally, the main therapies approved for the treatment of HCC patients, first- and second-line therapies, are described in this review. We also describe a novel option, pirfenidone, which due to its pharmacological properties could be considered in the future as a therapeutic option for HCC treatment.

## 1. Introduction

Liver cancer is the fourth main cause of cancer-related death worldwide, and according to estimations of the World Health Organization, more than one million people will die from this disease in 2030 [1].

The main risk etiological factors for liver cancer are infections by C or B hepatitis virus, chronic alcohol consumption, and, in the last years, nonalcoholic fatty liver disease (NAFLD); however, smoking, obesity, diabetes, and iron overload have been factors implicated in the generation of this disease [2].

Numerous cellular mechanisms as dysregulation of cell cycle and apoptosis, molecular pathways related with inflammation, and fibrogenesis processes are involved in liver cancer development; all these in turn represent important molecular targets for the development of novel drug therapies [3]. Regarding this, the first-line therapy for advanced HCC is sorafenib; a multikinase inhibitor approved for liver cancer treatment; this drug has been demonstrated to provide a significant improvement in the overall survival but is unable to counteract the disease progression due to the development of resistance to antiproliferative therapies [4]. Therefore, it is urgent to developing new molecules with pharmacological efficacy and safety.

Animal models have played an important role in biomedical research and are a crucial tool for study and understanding the pathogenesis of several liver diseases, including cancer; they also help to evaluate the pharmacological efficacy and safety of new drugs [5]. A wide range of experimental liver cancer models are available, and each one has its limitations and scopes; the choice of one of them depends on the aims established. An ideal animal model should mimic human liver cancer natural history, physiopathology, and biochemistry [6].

The aim of this work is provide a review about the current drugs approved for liver cancer treatment, its advantages and limitations, cell signal pathways that participate in cancer pathogenesis, and the main cell targets to which these therapies are aimed. Finally, we will review the most used experimental models for the study of this disease, including its methodological basis, their similarities with the human disease, and its main characteristics.

## 2. Molecular Targets and Signal Pathways Related With HCC Development

**Figure 1:**
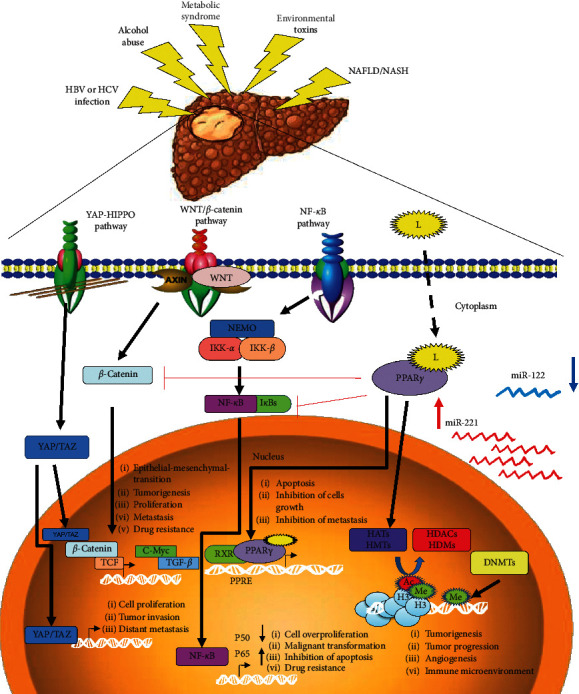
Main mechanisms involved in the development of hepatocarcinoma. The chronic exposure of hepatoxic agents causes the activation of the signaling pathways. YAZ-HIPPO receptors regulate the YAZ/TAZ transcriptional dimer genes involved in increased cell proliferation, mesenchymal epithelial transition, and metastasis. On the other hand, WNT/AXIN regulates the translocation of *β*-catenin to the nucleus, forming the YAZ/TAZ-*β*-catenin-TCF trimer activating profibrotic factors such as TGF-*β* and proliferative factors such as C-Myc, contributing to tumorigenesis and metastasis and inducing resistance to anticancer drugs. Similarly, NF−*κ*B signaling pathway and the dimers IKK-*α* and IKK-*β* induce differential translocation of NF−*κ*B P50/P65; on the one hand, an increase of P65 is induced to the nucleus while p50 is not translocated, causing an increase in cell proliferation, inhibition of apoptosis, transformation of malignant cells, and contributing to drug resistance. Finally, PPAR*γ* bound to its ligands has a dual effect, inhibiting the signaling pathway of *β*-catenin and NF-*κ*B, but by binding to the PPRE region it can increase the expression of genes involved in apoptosis, inhibition of cell proliferation, and metastasis. Acetylation and methylation of histones, regulated by the HATs/HDACs and HMTs/HDMs balance and DNA methylation regulated by DNMT1 activity, are the main epigenetic marks associated with tumorigenesis.

Several cellular signaling pathways implicated in hepatocarcinogenesis such as Yes-Associated Protein-Hippo Pathway (YAP-HIPPO), Wnt/*β*-catenin and NF-*κ*B have been studied. Additionally, the regulation of lipid metabolism by PPAR*γ*, the participation of epigenetic changes such as acetylation and methylation of histones, DNA methylation, and noncoding RNAs have been recently proposed such as important processes in the development of HCC. In the next sections, important characteristics of each cellular signaling pathway as well as their role in liver carcinogenesis will be debated. In Figure 1, we showed a detailed description of these pathways related with HCC pathology.

### 2.1. Yes-Associated Protein- (YAP-) Hippo

YAP1 is a protein that acts as a transcriptional regulator of genes involved in cell proliferation and suppressing apoptotic genes. *YAP1* is an oncogene in various human cancers [7]. When the pathway is activated, yes-associated protein 1 (YAP1) and its paralog transcriptional coactivator with PDZ-binding motif (TAZ) are phosphorylated on a serine 314 residue and sequestered in the cytoplasm by 14-3-3 proteins. In the opposite direction, when Hippo pathway is inactivated, YAP1/TAZ enters the nucleus and regulates gene expression [7].

Hippo-YAP pathway regulates the size of liver cells and proliferation, apoptosis, invasion, and metastasis of hepatoma cells [8]. In the liver of transgenic mouse, YAP overexpression results in a 2-fold increase in the mass of this tissue since the first week after birth; when YAP expression returned to normal levels, liver tumors were significantly reduced, and the liver parenchyma gradually returned to normal [9].

Varelas et al. demonstrated that Hippo-Yap pathway negatively regulates the Wnt/*β*-catenin pathway and that loss of TAZ increases Dishevelled segment polarity protein 2 (DVL2) phosphorylation and Wnt/*β*-catenin activation, as shown in enhanced cytoplasmic and nuclear *β*-catenin in cellular cultures and in kidneys of *Taz*-null mice [10]. These reports propose that YAP/TAZ can act such as either oncogene or tumor suppressor depending on a given stimulus. On the other hand, YAP-HIPPO signaling pathway acts such as an important point of regulation for liver tumors formation and some molecular intermediaries as new pharmacological targets in the design of new therapeutic strategies.

### 2.2. WNT/*β*-Catenin Signaling

Beta-catenin is a functional protein that has activities according to its cellular location. In cellular membrane, it is a component of a binding complex with cadherins to maintain the cytoskeleton structure; it also participates in cellular signaling and cellular adhesion [11]. In nucleus, *β*-catenin is a downstream effector of Wnt/AXIN complex that functions as a cofactor for T-cell factor/lymphoid enhancer factor (TCF/LEF) transcriptional factors [12].

During HCC, *β*-catenin signaling pathway is overactivated and allows promoting the proliferation of target genes such as c-Myc and cyclin D1 [13]. Oncogenic activation may be due to the inactivation of tumor suppressor adenomatous polyposis *coli* (APC), by direct mutation of *β*-catenin, which prevents the proteasomal degradation of this protein [14]. Furthermore, oncogenic activation of *β*-catenin in combination with mutations in genes such as ARID2, NFE2L2, TERT, APOB, and MLL2 or with the activation of hepatocyte growth factor receptor (HGFR or c-MET) can help to develop tumorigenesis [15].

### 2.3. NF-*κ*B Pathway

Chronic inflammation, tissue remodeling, genetic modifications, and alterations in cellular signaling are considered key processes implicated in the development and progression of HCC. NF-*κ*B is a transcription factor with the ability to regulate genes related with immune and inflammatory response [16]; its signaling pathway can be activated due to a chronic inflammation and the subsequent proinflammatory cytokines production, which can be potentially harmful for the liver [17]. In the canonical pathway, NF-*κ*B is inhibited by I*κ*B protein; when I*κ*B is phosphorylated in Ser32 and Ser36 by kinase complex (IKK), I*κ*B is degraded by proteasomal complex dependent of ubiquitin, allowing NF-*κ*B entry into nucleus, binding to DNA, and beginning its transcriptional activity [17, 18].

Several studies not only emphasize the key role of NF-*κ*B in the progression of liver disease processes, but also highlight the links between liver injury, inflammation, fibrosis, and the development of HCC, mainly its association with apoptosis inhibition, cancer initiation, tumor cell proliferation, and tumor progression [17–19].

### 2.4. PPAR*γ* Activation

PPAR*γ* is a nuclear transcription factor, whose activation set up lipid metabolism, insulin sensitization of peripheral cells, and anti-inflammatory action. This nuclear factor is activated by binding its ligand, then heterodimerizing with retinoid *X* receptor, to finally bind with specific response elements in nucleus, called peroxisome proliferating response elements (PPRE) [20]. Activation of PPAR*γ* by agonists such as thiazolidinediones has been shown to have, *in vitro* and *in vivo,* anticancer effect in many cancer types reducing cell proliferation and preventing differentiation in cancer cells [20]. PPAR*γ* ligands, such as SR1664, showed efficacy to reduce type 1 collagen quantity and to prevent HSC activation, showing its antifibrotic properties in an animal model of liver damage, and ability to prevent HCC development [21].

### 2.5. Epigenetics

Hepatocarcinogenesis implicates genomic aberrations regulated by genetic and epigenetic modifications. HCC development is divided into early and late stages; interestingly, epigenetic regulation is involved in HCC development in both stages. The next section describes the main epigenetic modifications related with HCC development.

### 2.6. Histone Modifications

These processes are governed by enzymes which add and remove acetyl groups to histones. In a particular way, histone deacetylation is related with HCC pathogenesis through activity of histone deacetylase 3, which is an important factor that regulates HepG2 cells proliferation in an *in vitro* model [22, 23], along with histone deacetylase 6 which suppresses tumors through autophagic cell death [23, 24].

On the other hand, modifications such as histone 3 lysine 9 acetylation (H3K9) regulate the structure of histone and modulate transcriptional factors binding with target gene promoters. Human HCC cells (HepG2) in culture display a nucleosome density that is relatively lower than normal cells, in addition to H3K9 acetylation; indicating that H3K9 acetylation may play an important role in nucleosome relaxing and in tumorigenesis initiation [25]. Another study showed the importance of H3K9 acetylation that includes CBP/*p*300 analysis which has histone acetyltransferase (HAT) activity and is involved in many cellular processes. Results suggest that the decrease in CBP/*p*300 reduces the acetylation of H3K9, and this has an important role in malignant transformation, proliferation, apoptotic, and invasion in HCC [26].

### 2.7. DNA Methylation

DNA methylation modifications are common hallmarks in cancers, with more than 3000 hypomethylated promoters being identified in HCC tumor samples; genes more affected with these modifications are related with cell proliferation, adhesion, cell signaling, mobility, and invasion [27, 28]. Conversely, an important number of tumor suppressor genes hypermethylated in early stages of HCC have been observed [29].

CpG islands methylation is a typical DNA modification, and it is regulated by DNA methyltransferases (DNMTs). DNMT1 enzyme is involved in the configuration and regulation of tissue-specific cytosine methylation patterns. Aberrant methylation patterns have been associated with a great number of diseases, but mainly the development of various types of cancer. Diverse studies have linked the regulation of DNMT1 with HCC development [30]. Oh et al. were the first to characterize DNMT1 overexpression in human hepatocarcinoma tissue, and an increase in the methylation of genes such as p16, p15, E-cadherin, hypermethylated in cancer 1 (HIC-1), and Ras association domain family 1 isoform A (RASSF1A) was found correlating with a bad prognosis [31].

Different molecules that regulate DNMT1 activity have been used. 5′-Azacytidin, a prominent demethylating agent, has been tested, alone or in combination in human hepatocarcinoma cells, demonstrating a decrease in the activity of oncogenes and an increase in the proapoptotic pathways [32]. On the other hand, Ceccarelli et al. demonstrated that eicosapentaenoic acid (EPA), a fatty acid with anticancer properties, inhibits histone deacetylases (HDAC1) and DNMT1 expression and activity, thus promoting the expression of tumor suppressor genes. In hepatocarcinoma cells, EPA binds and activates PPAR*γ*, decreasing HDAC1 expression, which in turn decreases DNMT1 expression [33].

### 2.8. MicroRNAs (miRNAs)

MicroRNAs represent single-stranded 18–22 nucleotide-long noncoding RNAs with the ability to decrease the stability of translation of a number of messenger RNAs [28]. Regarding cancer development, a dysregulation of miRNAs leads to an abnormal expression of target genes, favoring progression, invasion, and metastasis [34]. MiRNAs mainly implicated with HCC development are miR-122 and miR-221 [35]. The physiological role of miR-122 is to maintain the identity of adult hepatocytes and preserve their normal physiological functions. During the initial phase of experimentally induced hepatocarcinogenesis, miR-122 is decreased. In HCC patients, low levels of miR-122 correlate with metastasis and poor prognosis [36]. The development of liver inflammation, steatohepatitis, fibrosis, and HCC has been observed in miR-122 KO mice [37]. On the other hand, miR-221 is an miRNA that is overexpressed in HCC and is related with early events of liver carcinogenesis and a poor prognosis. The activity of miR-221 is related to oncogenic cellular pathways modulation, mainly those related to cell proliferation, survival, and metastasis [35]. Lastly, miR-221 overexpression is also related with sorafenib resistance, as observed in HCC experimental models [38].

## 3. Experimental Models for HCC Research

The animal models describe and replicate the stages of human liver cancer development. Each model meets specific characteristics for the generation of tumors; however, differences between the time of development of HCC and the similarity with pathophysiology in early stages of human carcinoma allow some models to have advantage over others. For example, modified resistant hepatocyte model (MRHM) is a model of hepatocarcinogenesis that simulates the stages of initiation, promotion, and progression [39, 40]. This model is characterized by the initial administration (day zero) of diethylnitrosamine (DEN) by intraperitoneal route that causes DNA rupture and modifications in base sequence, followed by a consecutive oral administration of 2-acetylaminofluorene reducing normal hepatocytes growth (days 7, 8, and 9) and, finally, a partial hepatectomy (PH) of 75% of hepatic parenchyma that triggers hepatocytes growth modified by DEN (day 10) [39, 40]. Using this methodological strategy, it has been described that the first preneoplastic nodules appear after 30 days, solid tumors with physical appearance at the 5th month, and a tumor invasion at the 12th month. The advantages of this model make it possible to study the evolution of cancer from the very early to late events occurring in the natural history of human HCC disease and that are simulated by this model. In addition, a better reproducibility and an elevated incidence rate of tumor development among the study animals are achieved [39, 40]. On the other hand, there are only few models overlapping with cytotoxic application of chemicals inducing carcinogenic damage that show reproducibility, time of tumor formation, and HCC incidence. For example, DEN model is a commonly used model since it is effective in producing tumors with an incidence of 100% of the study subjects; this model generates fibrogenesis and loss weight in experimental animals and also allows the presence of inflammatory infiltrate and proinflammatory cytokines, yet a constant and consecutive weekly application of DEN is necessary for 15 weeks, starting from 5 weeks of age of the animals under study. It is important to mention that it is difficult to establish the onset of the appearance of carcinogenic damage and, therefore, the analysis of the events that occur in the early stages of the disease [41].

The CCl_4_ model allows HCC implementation through fibrosis and cirrhosis generation and the first neoplastic lesions that would become part of adenomas and carcinomas in different tissues; this model also offers the advantage of analyzing genotoxic events at the hepatic level and liver fat accumulation as well as chronic progressive nephropathy. However, a disadvantage is represented by the fact that the mortality rate is hasty and the constant administration of CCl_4_ for 104 weeks is necessary [42].

The choline deficiency-supplemented ethionine (CDE) diet model and the thioacetamide (TAA) supplementation model are models that allow the study of end-stage chronic liver disease and represent the same cellular events that occur in human liver disease regardless of the underlying cause or etiologic factor. These models are characterized by hepatocellular necrosis, chronic inflammation, fibrosis, proliferation of liver progenitor cells, and ductular reactions; while CDE or TAA is administered for a long time, neoplastic lesions will begin to appear and eventually turn into tumors. One disadvantage is the time required for tumor formation, which can range from 6 to 12 months. In addition, both experimental models do not allow the analysis of early HCC events, but they do allow a continuous flow of the disease to be followed where there is fibrosis, steatosis, cirrhosis, and HCC [43]. Finally, there are some models that simulate the dietary characteristics of an obesogenic environment and gradually lead to the development of HCC; among these models are high-fat diet with exposure to streptozotocin (STAM), American lifestyle-induced obesity syndrome (ALIOS) model, and diet-induced nonalcoholic fatty liver disease animal model (DIAMOND). These models allow the progressive accumulation of intrahepatic lipids for the generation of steatosis. In addition, some animals subjected to these models develop fibrosis. At the histological level, inflammatory infiltration and formation of fat droplets can be observed. Nonetheless, time and reproducibility are the main disadvantages since they are models that require constant administration of a diet high in fat and/or supplemented with cholesterol for approximately 12 months to promote tumor formation [44–46].

Table 1 describes a summary of several experimental models for HCC research, according to their cytotoxic, dietary, and genetic characteristics [47–49].

## 4. Pharmacological Systemic Drugs in HCC

HCC pathogenesis is related with cell cycle, apoptosis, and other important signal pathways dysregulation [3], because of this, several drugs used in HCC treatment focus their action on the blockade of some of these processes.

In a general way, the current pharmacological treatments employed for HCC patients are classified as first-line and second-line therapies. The most important pharmacological characteristics of these drugs are described as follows.

### 4.1. First-Line Therapies

#### 4.1.1. Sorafenib

It is the first drug approved for systemic treatment of patients with advanced HCC who are not candidates for liver transplantation or surgical resection. Sorafenib is an inhibitor of tyrosine kinase receptor (TKRs) related with angiogenesis, cellular differentiation, proliferation, and survival [50]. In a phase III clinical randomized controlled trial (SHARP), sorafenib reported an increase in 10.7 months' survival versus 7.9 months with placebo; the adverse events were diarrhea, fatigue, and hand–foot skin reaction [51]. Studies have shown evidence that sorafenib response is related with its ability to correct abnormal glycosylation in erythroblastosis 26-1 (Ets-1) protein in HCC cells, improving overall survival (OS) significantly but only in advanced HCC patients [3, 52].

#### 4.1.2. Lenvatinib

It is an effective drug that increases the OS in HCC patients whose tumor cannot be removed by surgery, reducing angiogenesis and lymphangiogenesis responses [53]. Phase I studies suggested that lenvatinib is effective in patients with advanced HCC and a Child–Pugh A or B score, 12 mg and 8 mg, respectively [54]. Hypertension, diarrhea, decreased appetite, and decreased weight were the main adverse events reported at a dose of 12 mg daily of oral lenvatinib [55].

### 4.2. Second-Line Therapies

#### 4.2.1. Regorafenib

This drug developed by Bayer was FDA-approved in June 2017 as a second-line oral drug for unresectable HCC [56]. Comparing regorafenib and sorafenib effects, the first drug has shown more effectiveness in inhibiting tyrosine kinases and phosphatases, with a better drug tolerance profile in HCC patients. Regarding survival, in patients treated with regorafenib, the median survival was 10.6 months, as compared to 7.8 months in the placebo group. Its main limitation is that only a few patients are eligible for this treatment, mainly by the deterioration of liver function [57].

Finally, sorafenib but not regorafenib treatment caused body weight loss and liver and kidney dysfunction, while regorafenib but not sorafenib treatment caused hypertension [57].

#### 4.2.2. Ramucirumab

This is a fully human anti-VEGFR-2 monoclonal antibody that blocks binding of the VEGFR ligands [58]. Its anticancer activity was observed in a phase II and III trial (REACH-2), in patients with advanced HCC and high levels of alpha-fetoprotein [59, 60]. This study showed an improved overall survival in patients treated with ramucirumab compared with placebo; this drug was well tolerated and with a controllable safety profile [60].

### 4.3. Future Potential Therapies

#### 4.3.1. Pirfenidone (PFD)

This is an antifibrotic, antioxidant, and anti-inflammatory drug, which has been evaluated in clinical and preclinical studies for the treatment of pulmonary and hepatic fibrosis [61–63]. This drug was effective in inducing G0/*G*1 cell cycle arrest in an *in vitro* model [64] and in inhibiting cell proliferation. Also, it promotes apoptosis of HepG2 cells through Wnt/*β*-catenin signaling pathway [65]. Additionally, PFD has shown to be a powerful antifibrotic drug at dose of 300 mg/kg in an experimental HCC model induced by carbon tetrachloride in mice [66], but the pharmacodynamic mechanisms involved in responses generated by PFD have yet to be clarified. It has recently been shown that pirfenidone can bind to ligand binding domain of PPAR*α*, which is a PPAR*γ* homolog, activating the SIRT1/L*κ*B1/pAMPK indicating their ability to modify the epigenetic marks of the H3K9 [67].

Table 2 summarizes some important pharmacological characteristics of each one of the previously described therapies.

## 5. Conclusions

HCC is the most common liver cancer, and its etiology is related with activation of multiple processes related with dysregulation of cell cycle, apoptotic response, and the activation of several signaling pathways that induce the inflammatory and fibrogenic response. Currently, patients with HCC have access to different therapeutic options, being the goals of all of them to improve liver function, survival, and quality of life of patients, but only a small number of these bioactive drugs have shown successful responses without causing side effects. Therefore, the use of experimental models represents a fundamental instrument in the development of new therapies for HCC treatment; in this way, the goal of future therapies should be to design novel pharmaceutical forms containing multiple drugs or to discover a single drug capable of modulating various signaling pathways related with HCC pathogenesis.

## Figures and Tables

**Table 1:** Pathophysiological characteristics between cytotoxic HCC experimental models.

Model	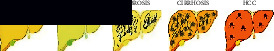					HCC time development	Highlights	Ref
MRHM	+	+	+	−	+	5–12 months	Infiltration, preneoplasia at 30 days, high number of positive fields for GGT. Fibrosis, inflammation, metabolic alterations. Allows the study of early and late HCC.	[39, 40]
DEN	+	−	−	−	+	9 months	Chronic inflammation, chromosomal instability, disruption of cell cycle, DNA damage, neutrophil infiltration, bile duct proliferation, centrilobular hemorrhagic necrosis, bridging necrosis.	[41]
CCl_4_	+	+/−	+	−	+	1-2 years	Hepatic collagen accumulation, periportal fibrosis, hepatocyte necrosis, stellate hepatic cell activation, macrophage infiltration.	[42]
TAA	+	+/−	+	+	+	6–12 months	Strong centrally driven fibrotic component, progressing to cirrhosis prior to HCC.	[43]
STAM	+	+	+	−	+	20 weeks	Hepatic fat deposit whilst increased lobular inflammation with foam cell-like macrophages.	[44]
CDE	+	+/−	+/−	−	+	14 months	Major steatosis, periportal injury, fibrosis, strong liver progenitor cell component.	[43]
ALIOS	+	+/−	+/−	−	+	1 year	Ballooning hepatocytes, fibrosis, later liver progenitor cell involvement.	[45]
DIAMOND	+	+/−	+	−	+	1 year	Ballooning and progressive fibrosis. Strong histologic and transcriptomic similarities with human NASH and HCC.	[46]
DEN + HFD	+	+	+	+	+	9 months	Characterized model of enhancing IL-6 and TNF expression.	[47]
DEN + CCl_4_	+	+/−	+	−	+	5–9 months	Inflammation, fibrogenesis, oxidative stress, shortened HCC latency, and increased presence of progenitor cells.	[48, 49]

^+^Present;  ^−^absent;  ^+/−^may or may not develop in experimental models (relative incidence). DEN: diethylnitrosamine; CCl_4_: carbon tetrachloride; MRHM: modified resistant hepatocyte model; TAA: thioacetamide; STAM: streptozotocin + HFD-treated mice; CDE: choline-deficient ethionine-supplemented diet; ALIOS: American lifestyle-induced obesity syndrome model; DIAMOND: diet-Induced animal model of nonalcoholic fatty liver disease; and HFD: high-fat diet.

**Table 2:** Main pharmacological characteristics of systemic drugs used in HCC treatment.

Drug	Dose	Family	Molecular target	Reference
First-line drugs				
*Sorafenib*	800 mg/day	Inhibitors of tyrosine kinase receptors (TKRs)	VEGFR-1, VEGFR-2, VEGFR-3, PDGFR*β*, RET, c-KIT, and FMS-like tyrosine kinase-3, Ras/MAPK pathway, and wild-type and mutant Raf-1 (C-Raf) and B-Raf	[50, 51]
*Lenvatinib*	12 mg/day	Inhibitors of TKRs	VEGFR, FGFR 1–4, PDGFR, and SCF	[53–55]
Second-line drugs				
*Regorafenib*	160 mg/day	Inhibitors of TKRs	VEGFR-1, VEGFR-2, VEGFR-3, PDGFR*β*, FGFR-1, KIT, RET, RAF1, and BRAF	[56, 57]
*Ramucirumab*	8 mg/kg every 2 weeks	Human monoclonal antibody (IgG1)	VEGFR-2	[58–60]
Futures therapies				
*Pirfenidone*	300–600 mg/Kg (mouse model)	Pyridones	Induction G0/*G*1 cycle arrest, inhibition of Wnt/*β*-catenin signaling pathway, ligand/activator of PPAR*α* and PPAR*γ*; SIRT1/L*κ*B1/pAMPK activation	[65–67]

TKR: tyrosine kinase receptors; VEGFR: vascular endothelial growth factor receptor; PDGFR: platelet-derived growth factor receptor; FGFR: fibroblast growth factor receptor; SCF: steam cell factor; EGF: epidermal factor receptor; PPAR*α*: peroxisome proliferator-activated receptor alpha; PPAR*γ*: peroxisome proliferator-activated receptor gamma; *α*-SMA: alpha-smooth muscle actin; TGF-*β*: transforming growth factor-beta; TNF-*α*: tumor necrosis factor-alpha; COX-2: cyclooxygenase 2; PCNA: proliferating cell nuclear antigen; NF-*κ*B: nuclear factor-*κ*B; SIRT1: sirtuin 1; L*κ*B1: liver kinase B1; and pAMPK: phospho-AMP-activated protein kinase.

## Data Availability

The data used to support this study are included within article as references.
